# Identifying key risk factors for acute compartment syndrome in tibial diaphysis fracture patients

**DOI:** 10.1038/s41598-024-59669-1

**Published:** 2024-04-17

**Authors:** Ming An, Ruili Jia, Limei Wu, Leilei Ma, Hui Qi, Yubin Long

**Affiliations:** 1 Department of Orthopedics, The First Central Hospital of Baoding, Baoding, 071000 China; 2 Department of Nephrology, The First Central Hospital of Baoding, Baoding, 071000 China; 3 Hebei Provincial Hospital of Traditional Chinese Medicine, Shijiazhuang, 050000 China; 4 Department of Burns and Plastic Surgery, The Third Hospital of Hebei Medical University, Shijiazhuang, 050000 China

**Keywords:** Tibial diaphysis fractures, Acute compartment syndrome, Crush injury, Lactic dehydrogenase, White blood cell, Cholinesterase, Trauma, Risk factors

## Abstract

Acute compartment syndrome (ACS) is a severe orthopedic issue that, if left untreated, can result in lasting nerve and muscle damage or even necessitate amputation. The association between admission laboratory blood test indicators and the occurrence of ACS in patients with tibial diaphysis fractures is currently a subject of debate. The objective of this research was to identify the contributing factors for ACS in individuals suffering from tibial diaphysis fractures. In this retrospective study, we collected data on a total of 705 individuals from our hospital, comprising 86 ACS patients and 619 non-ACS patients with tibial diaphysis fractures. These participants were categorized into two distinct groups: the ACS group and the non-ACS group. Despite the inherent limitations associated with retrospective analyses, such as potential biases in data collection and interpretation, we conducted a comprehensive analysis of demographics, comorbidities, and admission lab results. Our analytical approach included univariate analysis, logistic regression, and receiver operating characteristic (ROC) curve analysis techniques, aiming to mitigate these limitations and provide robust findings. The statistical analysis revealed several predictors of ACS, including gender (p = 0.011, OR = 3.200), crush injuries (p = 0.004, OR = 4.622), lactic dehydrogenase (LDH) levels (p < 0.001, OR = 1.003), and white blood cell (WBC) count (p < 0.001, OR = 1.246). Interestingly, the study also found that certain factors, such as falls on the same level (p = 0.007, OR = 0.334) and cholinesterase (CHE) levels (p < 0.001, OR = 0.721), seem to provide a degree of protection against ACS. In order to better predict ACS, the ROC curve analysis was employed, which determined threshold values for LDH and WBC. The established cut-off points were set at 266.26 U/L for LDH and 11.7 × 10^9^ cells per liter for WBC, respectively. Our research has successfully pinpointed gender, crush injuries, LDH levels, and white blood cell (WBC) count as crucial risk factors for the development of ACS in patients experiencing tibial diaphysis fractures. Furthermore, by establishing the cut-off values for LDH and WBC, we have facilitated a more personalized assessment of ACS risk, enabling clinical doctors to implement targeted early interventions and optimize patient outcomes.

## Introduction

Tibial diaphysis fractures are among the most common long bone injuries affecting the general population, with an incidence rate of 16.9 per 100,000 individuals annually^1,2^. Males experience a higher occurrence of these fractures, at a rate of 21.5 per 100,000 people per year, particularly within the age range of 10 to 20 years. In contrast, females display a lower frequency of 12.3 per 100,000 people per year, with the highest incidence noted between the ages of 30 and 40^3^. Among the various fracture types, the Arbeitsgemeinschaftfür Osteosynthesefragen (AO) classification type 42-A3 is identified as the most prevalent in tibial diaphysis fractures^4^. The leading causes of diaphyseal fractures are falls on the same level, sports-related activities, and road traffic accidents^5,6^. Patients who have sustained tibial diaphyseal fractures may encounter tenderness upon palpation of the fracture site. If there is considerable disruption of the periosteum and displacement, a discernible clinical deformity might also be evident. Additionally, when the pressure within the closed compartments of the lower limb rises (including the anterior, lateral, superficial posterior, and deep posterior compartments), there is a possibility of developing acute compartment syndrome (ACS) ^7^. It is worth noting that tibial diaphyseal fractures stand as the leading cause of ACS, with an estimated 36% of ACS cases being attributed to these specific fractures^8,9^.

Acute compartment syndrome (ACS) in the lower leg poses a significant threat to limb integrity and demands immediate surgical intervention. This condition occurs when increased pressure within a muscle compartment, enclosed by an inelastic fascial space, leads to reduced tissue perfusion, ultimately compromising motor and sensory functions within the affected area^8,10,11^. If timely intervention is not provided, the elevated pressure in muscle tissue, diminished blood flow, and compromised tissue oxygenation can result in prolonged hospital stays, increased expenses, and potentially poor outcomes, such as amputation and even death^12^. Hence, gaining a thorough understanding of the characteristics of ACS is essential for grasping its onset and progression. Numerous prior studies have pinpointed various risk factors associated with the development of ACS in patients with tibial fractures, including younger age, male gender, individuals without a history of hypertension, high-energy injuries, absence of hypertension, and the presence of fibular fractures^13–16^. Nonetheless, the role of admission laboratory indicators in predicting ACS among patients with tibial diaphyseal fractures has received limited attention. Consequently, gaining a comprehensive understanding of the severity of this issue remains a challenge.

The objective of this study is to explore the associations between the development of ACS in patients with tibial diaphyseal fractures and a variety of demographic factors, comorbidities, and notably, admission laboratory assessments, which may serve as potential predictors for the onset of ACS. A thorough comprehension of these risk factors and their individual impacts on the development of ACS can enable the identification of high-risk patients, which is essential for improving patient outcomes.

## Methods

### Ethics statement

We performed an extensive retrospective assessment on a cohort of 705 patients who had been diagnosed and treated for diaphyseal tibial fractures, with or without ACS, at Baoding First Central Hospital. The study, which spanned from November 2013 to January 2021, received approval from the hospital's institutional review board (Ethics Committee of Baoding First Central Hospital) and adhered to the ethical principles set forth in the 1964 Helsinki Declaration (2022116). The study was based on the electronic medical records of these patients. In light of our study being a retrospective research, The Ethics Committee of Baoding First Central Hospital agreed to our right not to obtain informed consent from each patient included.

### Patients

**Figure 1:**
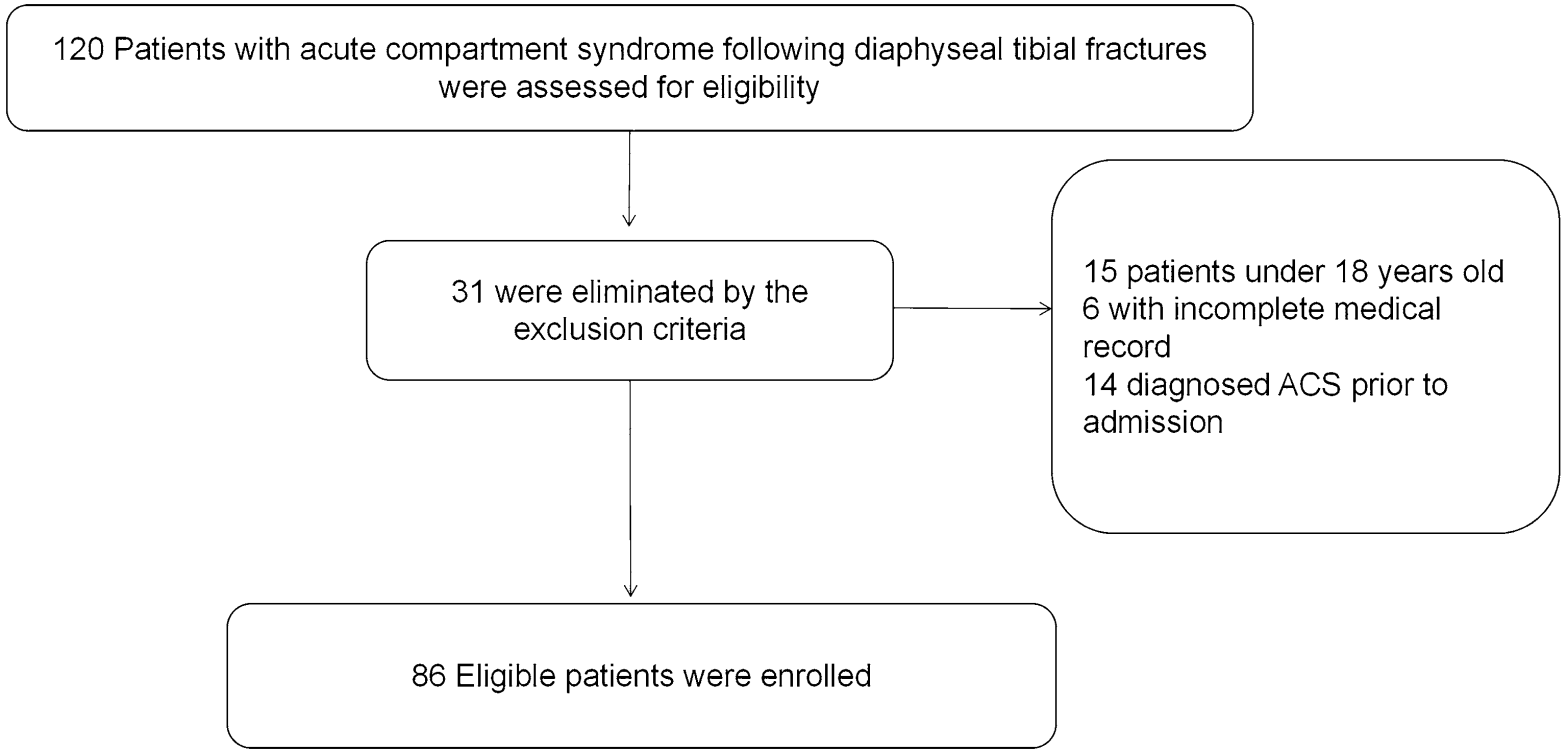
Flow diagram of included patients.

The research was conducted at our hospital, a distinguished tertiary institution equipped with a level I trauma center. As a retrospective study, our approach involved scrutinizing historical medical records to collect pertinent data. We defined exclusion criteria for this investigation, which covered patients under 18 years old, those who diagnosed ACS prior to admission, and cases with incomplete medical documentation. Consequently, after implementing these criteria, our study encompassed a total of 705 patients, consisting of 514 men and 191 women (Fig. 1).

Among the 705 patients, 86 experienced ACS following diaphyseal tibial fractures, whereas the remaining 619 patients, sustained the same type of fractures but did not develop ACS. To facilitate comparison, we divided the patients into two distinct groups according to the presence or absence of ACS after their diaphyseal tibial fractures: the ACS group and the non-ACS group.

The presentation of diaphyseal tibial fractures may be diverse, influenced by the severity and underlying cause of the injury. However, common symptoms experienced by most patients include pain, discomfort, edema, and difficulty in bearing weight. Radiographic assessment should involve anterior and posterior plain radiographs of the tibia, encompassing both the knee and ankle to minimize unnecessary radiation exposure. Furthermore, it is essential to meticulously examine the hip, femur, and vascular structures to exclude the possibility of concurrent injuries. Surgical intervention becomes necessary when encountering open fractures, fractures occurring in the context of polytrauma, floating knee injuries, certain segmental fractures, vascular injuries, unstable fractures where adequate alignment cannot be achieved or maintained, and some fractures accompanied by severe swelling and concerns for ACS^17^. The diagnosis of ACS primarily relies on physical examination and the recognition of six fundamental clinical signs, often referred to as the six P's. These encompass: (1) pain, (2) poikilothermia, (3) paresthesia, (4) paralysis, (5) pulselessness, and (6) pallor^8,11,18,19^. Every patient with ACS would experience increased pain with passive extension in the early stages of the disease. However, the latter five symptoms are late-stage manifestations of ACS and may not be present in all cases. To diagnose ACS in patients with less obvious clinical manifestations of ACS, we employ direct measurement of muscle tissue pressure using established criteria. These patients undergo regular clinical evaluations and hourly monitoring of intracompartmental Pressure (ICP) before and after their surgeries, which typically span 24 to 48 h. A continuous ICP slit catheter is inserted, and our nursing staff takes hourly measurements. Any unusual clinical findings or abnormal pressure readings promptly alert the surgical team. A sustained ΔP, which is the difference between diastolic blood pressure and compartment pressure, less than 30 mm Hg for 2 h is considered diagnostic of ACS. This method of tissue pressure measurement is both objective and reproducible, reducing the impact of subjective judgment and diagnostic errors. In addition to diagnosing based on clinical presentation, we also used pressure measurement as a more objective method. When clinical symptoms are not typical but ΔP < 30 mmHg (where ΔP = diastolic blood pressure—compartment pressure), the diagnosis of ACS can still be established. For patients with diaphyseal tibial fractures who are diagnosed with ACS, it is crucial to swiftly carry out a fasciotomy. This procedure alleviates pressure and reestablishes blood flow to the impacted compartment, thereby helping to avert adverse outcomes^20^. Diagnoses and surgical interventions were performed by seasoned orthopedic surgeons who held associate professor positions or higher and possessed a minimum of ten years of expertise in their respective fields.

This study collected data on patients, taking into account demographics, comorbidities, and notably, admission laboratory exams which have not been previously discussed. Demographics encompassed age, gender, BMI, referral, mechanism of injury, open and multiple fractures, history of tibial diaphysis fractures, smoking and alcohol history, and the use of dehydrating agents. Comorbidities such as arrhythmia, coronary heart disease, hypertension, diabetes, anemia, and hypoproteinemia were also examined. Furthermore, the study investigated an array of admission laboratory indicators, including WBC (white blood cell), NEU (neutrophil), LYM (lymphocyte), MON (monocyte), EOS (eosinophil), BAS (basophil), RBC (red blood cell), HGB (hemoglobin), HCT (hematocrit), MCV (mean corpuscular volume), MCH (mean corpuscular hemoglobin), MCHC (mean corpuscular hemoglobin concentration), PLT (platelet), MPV (mean platelet volume), DBIL (direct bilirubin), GGT (gamma-glutamyl transpeptidase), TP (total protein), ALB (albumin), GLOB (globulin), UREA (ureophil), UA (uric acid), LDH (lactic dehydrogenase), CK (creatine kinase), TG (triglyceride), TCO_2_ (total carbon dioxide), CHE (cholinesterase), ALP (alkaline phosphatase), and OSM (osmotic pressure).

### Statistical analysis

Data analysis was conducted utilizing SPSS software (version 25.0, SPSS Inc., New York, USA), and we deemed a p-value < 0.05 as statistically significant. We evaluated continuous variables for normality with the Shapiro–Wilk test. Normally distributed variables were presented as mean ± SD and analyzed using the t-test, while non-normally distributed variables were examined through the Mann–Whitney U test. For categorical variables expressed as counts and percentages, we assessed between-group differences employing the Chi-square and Fisher's exact tests. Ultimately, binary logistic regression analysis was utilized to pinpoint the independent risk factors for ACS among the patients.

Frequently, receiver operating characteristic (ROC) analysis is employed to establish optimal cut-off values following the peak of the Youden index. These values are then divided into low-risk and high-risk categories. To evaluate the diagnostic performance, we calculated the area under the ROC curve (AUC); a larger area signifies greater diagnostic accuracy.

### Ethics approval and consent to participate

This retrospective study was approved by the Institutional Review Board of our hospital (Ethics Committee of Baoding First Central Hospital) (2022116) before collecting data. There is no need to write informed consent forms from patients because this is a retrospective study.

## Results

This study involved 705 participants, including 514 males and 191 females, with 86 individuals developing ACS and the remaining 619 not exhibiting ACS. Among these 86 ACS patients, 76 were male and 10 were female. Therefore, in our study, the probability of female patients developing ACS was 5.15%, while for male patients it was 15.21%. Data from Table 1 disclosed notable disparities between the ACS and non-ACS groups concerning gender (p = 0.001) (indicating a higher likelihood of males developing ACS), referral patients (p = 0.001), patients with open fractures (p < 0.001), and injury mechanisms (p < 0.001). The ACS group exhibited a significantly elevated proportion of patients with crush injuries (p < 0.001) and a reduced proportion of patients experiencing falls on the same level (p < 0.001). In contrast, no substantial differences emerged between the two groups concerning age, BMI, multiple fractures, history of tibial diaphysis fractures, smoking and alcohol history, or the usage of dehydrating agents (all p > 0.05).

**Table 1:** Demographics data of patients with and without ACS.

Demographics	ACS group (n = 86)	Non-ACS group (n = 619)	*p*
Age, years	32.5 (24.8–53.0)	43.0 (30.0–56.0)	0.004*
Gender, n (%)			< 0.001*
Male	80 (88.9%)	446 (70.8%)	
Female	10 (11.1%)	184 (29.2%)	
BMI, kg/m^2^	24.3 (22.5–26.0)	24.1 (22.9–26.0)	0.223
< 24	10 (11.1%)	76 (12.1%)	0.619
24–28	51 (56.7%)	322 (51.2%)	
> 28	29 (32.2%)	231 (36.7%)	
Referral, n (%)			0.001*
Yes	73 (81.1%)	396 (62.9%)	
No	17 (18.9%)	234 (37.1%)	
Mechanism of injury, n (%)			< 0.001*
Car crash injury	31 (34.4%)	207 (32.9%)	
Falls on the same level	14 (15.6%)	292 (46.3%)	
Crush injury	12 (13.3%)	21 (3.3%)	
Hurt by a heavy object	12 (13.3%)	69 (11.1%)	
Unknown trauma	21 (23.3%)	41 (6.5%)	
Crush injury, n (%)			< 0.001*
Yes	12 (13.3%)	21 (3.3%)	
No	78 (86.7%)	609 (96.7%)	
Falls on the same level, n (%)			< 0.001*
Yes	14 (15.6%)	292 (46.3%)	
No	76 (84.4%)	338 (53.7%)	
Open fractures, n (%)			< 0.001*
Yes	29 (32.2%)	57 (9.0%)	
No	61 (67.8%)	573 (91.0%)	
Multiple fracture, n (%)			0.132
Yes	38 (42.2%)	215 (34.1%)	
No	52 (57.8%)	415 (65.9%)	
History of tibial diaphysis fractures, n (%)			0.516
Yes	3 (3.3%)	14 (2.2%)	
No	87 (96.7%)	616 (97.8%)	
Smoking history, n (%)			0.366
Yes	15 (16.7%)	83 (13.2%)	
No	75 (83.3%)	547 (86.8%)	
Alcohol history, n (%)			0.428
Yes	10 (11.1%)	54 (8.6%)	
No	80 (88.9%)	576 (91.4%)	
Whether to use dehydrating agent, n (%)			0.412
Yes	61 (67.8%)	399 (63.3%)	
No	29 (32.2%)	231 (36.7%)	

Values are presented as the number (%) or the median (interquartile range).

*BMI* body mass index.

*p < 0.05, statistical significance.

Table 2 highlighted the contrast in comorbidities between the ACS and non-ACS groups. A higher prevalence of hypoproteinemia (p < 0.001) and anemia (p = 0.007) was observed in the ACS group, whereas no significant differences were detected in other assessed comorbidities, such as arrhythmia, coronary heart disease, hypertension, and diabetes (all p > 0.05). Table 3 illustrated the laboratory findings at admission for both ACS and non-ACS groups. The data revealed that the ACS group had notably increased levels of WBC (p < 0.001), NEU (p < 0.001), MON (p < 0.001), EOS (p < 0.001), LDH (p < 0.001), and CK (p < 0.001) compared to the non-ACS group. In contrast, the non-ACS group presented relatively higher levels of ALB (p < 0.001), GLOB (p = 0.036), CHE (p < 0.001), Ca (p < 0.001), Na (p < 0.001), OSM (p = 0.029), TCO2 (p = 0.004), and TP (p < 0.001) than the ACS group. No significant differences were identified in other laboratory parameters between the two groups (all p > 0.05).

**Table 2:** Comorbidities data of patients with and without ACS.

Comorbidities	ACS group (n = 86)	Non-ACS group (n = 619)	*p*
Arrhythmia, n (%)			0.540
Yes	1 (1.1%)	13 (2.1%)	
No	89 (98.9%)	617 (97.9%)	
Coronary heart disease, n (%)			0.720
Yes	3 (3.3%)	26 (4.1%)	
No	87 (96.7%)	604 (95.9%)	
Hypertension, n (%)			0.079
Yes	7 (7.8%)	92 (14.6%)	
No	83 (92.2%)	538 (85.4%)	
Diabetes, n (%)			0.259
Yes	3 (3.3%)	40 (6.3%)	
No	87 (96.7%)	590 (93.7%)	
Anemia,n (%)			0.003
Yes	41 (45.6%)	190 (30.2%)	
No	49 (54.4%)	440 (69.8%)	
Hypoproteinemia,n (%)			< 0.001*
Yes	18 (20.0%)	17 (2.7%)	
No	72 (80.0%)	613 (97.3%)	

Values are presented as the number (%) or the median (interquartile range).

*p < 0.05, statistical significance.

**Table 3:** Laboratory results of patients with and without ACS.

Laboratory results	ACS group (n = 86)	Non-ACS group (n = 619)	*p*
WBC	13.35 (9.71–19.07)	9.15 (7.51–10.15)	< 0.001*
NEU	10.97 (6.80–16.18)	6.43 (5.03–8.32)	< 0.001*
LYM	1.62 (1.10–2.18)	1.39 (1.09–1.77)	0.022
MON	0.93 (0.62–1.15)	0.65 (0.50–0.81)	< 0.001*
EOS	0.11 (0.03–0.17)	0.04 (0.01–0.09)	< 0.001*
BAS	0.03 (0.01–0.07)	0.02 (0.02–0.04)	0.076
RBC	3.92 (3.45–4.59)	4.07 (3.75–4.47)	0.208
HGB	121.24 (110.38–140.70)	124.57 (115.55–138.20)	0.188
HCT	36.06 (32.46–41.01)	37.05 (34.55–40.71)	0.155
MCV	92.56 (89.07–96.60)	92.03 (88.29–95.24)	0.270
MCH	31.41 (30.08–32.51)	31.00 (29.60–32.19)	0.069
MCHC	336.90 (330.95–344.23)	335.80 (329.28–342.50)	0.138
PLT	209.50 (157.75–259.25)	220.70 (174.08–233.48)	0.770
MPV	8.64 (7.91–9.47)	8.68 (8.00–9.50)	0.777
DBIL	5.10 (3.15–6.67)	4.90 (3.40–6.61)	0.744
GGT	20.00 (13.00–31.31)	21.00 (15.00–35.25)	0.389
TP	57.28 (51.35–66.55)	64.23 (59.89–68.04)	< 0.001*
ALB	35.95 (31.68–41.56)	41.05 (38.08–44.30)	< 0.001*
GLOB	21.84 (18.55–25.03)	22.84 (20.02–25.43)	0.036*
UREA	5.24 (4.11–6.07)	4.93 (3.99–6.10)	0.568
UA	302.50 (241.75–395.50)	293.00 (226.75–380.25)	0.146
LDH	511.30 (233.00–809.16)	200.00 (170.55–240.75)	< 0.001*
CK	792.00 (310.25–3018.13)	282.25 (140.73–591.25)	< 0.001*
TG	1.18 (0.58–1.49)	1.04 (0.69–1.51)	0.920
TCO2	23.54 (22.00–26.00)	25.00 (22.90–27.00)	0.001*
CHE	6.34 (5.08–7.68)	7.67 (6.40–9.09)	< 0.001*
ALP	66.00 (50.75–81.00)	65.00 (53.00–80.00)	0.849
K	3.95 (3.65–4.20)	3.91 (3.66–4.20)	0.896
Na	137.47 (135.08–139.78)	139.13 (137.38–141.00)	< 0.001*
Cl	103.83 (101.17–106.93)	104.19 (102.20–106.20)	0.559
Ca	2.14 (1.97–2.26)	2.21 (2.12–2.32)	< 0.001*
P	1.13 (0.96–1.28)	1.13 (0.97–1.29)	0.801
OSM	270.35 (264.85–274.83)	270.70 (267.20–275.20)	0.098

Values are presented as the number (%) or the median (interquartile range).

*WBC* white blood cell, *NEU* neutrophil, *LYM* lymphocyte, *MON* monocyte, *EOS* eosinophil, *BAS* basophil, *RBC* red blood cell, *HGB* hemoglobin, *HCT* hematocrit, *MCV* mean corpuscular volume, *MCH* mean corpusular hemoglobin, *MCHC* mean corpusular hemoglobin concentration, *PLT* platelet, *MPV* mean platelet volume, *DBIL* direct bilirubin, *GGT* gamma glutamyl transpeptidase, *TP* total protein, *ALB* albumin, *GLOB* globulin, *UREA* ureophil, *UA* uric acid, *LDH* lactic dehydrogenase, *CK* creatine kinase, *TG* triglyceride, *TCO*_*2*_ total carbon dioxide, *CHE* cholinesterase, *ALP* alkaline phosphatase, *OSM* osmotic pressure.

*p < 0.05, statistical significance.

In this study, logistic regression analysis revealed significant associations between tibial diaphysis fractures and the development of ACS in male patients (p = 0.011, OR = 3.200, 95% CI (1.312 to 7.807)). Elevated WBC counts (p < 0.001, OR = 1.246, 95% CI (1.157 to 1.341)), elevated LDH levels (p < 0.001, OR = 1.003, 95% CI (1.002 to 1.004)), and a history of crush injury (p = 0.004, OR = 4.622, 95% CI (1.626 to 13.139)) were also found to be significantly associated with the development of ACS. On the other hand, a history of falls on the same level (p = 0.007, OR = 0.334, 95% CI (0.152 to 0.737)) and higher levels of CHE (p < 0.001, OR = 0.721, 95% CI (0.608 to 0.854)) were identified as protective factors against ACS in these patients (Table 4).

**Table 4:** Binary logistic regression analysis of variables associated with ACS.

Characteristics	OR	95% CI	*P*
Age	0.984	0.967 to 1.002	00.076
Gender	3.200	1.312 to 7.807	0.011*
Falls on the same level	0.334	0.152 to 0.737	0.007*
Crush injury	4.622	1.626 to 13.139	0.004*
LDH	1.003	1.002 to 1.004	< 0.001*
CHE	0.721	0.608 to 0.854	< 0.001*
WBC	1.246	1.157 to 1.341	< 0.001*

*LDH* lactic dehydrogenase, *CHE* cholinesterase, *WBC* white blood cell.

*p < 0.05, statistical significance.

**Figure 2:**
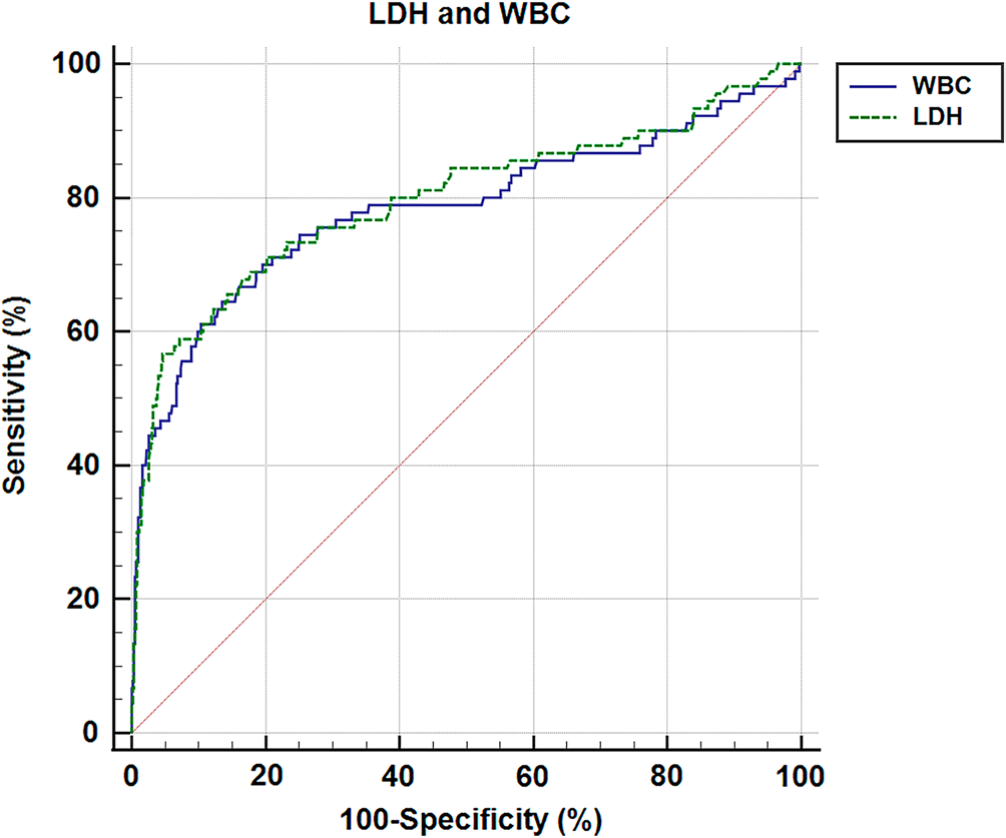
Comparison of ROC curves for LDH and WBC.

**Figure 3:**
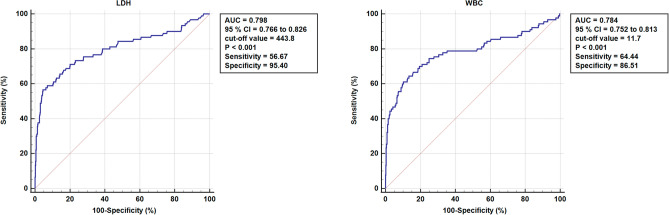
Detailed information on ROC curves separately for LDH and WBC.

**Figure 4:**
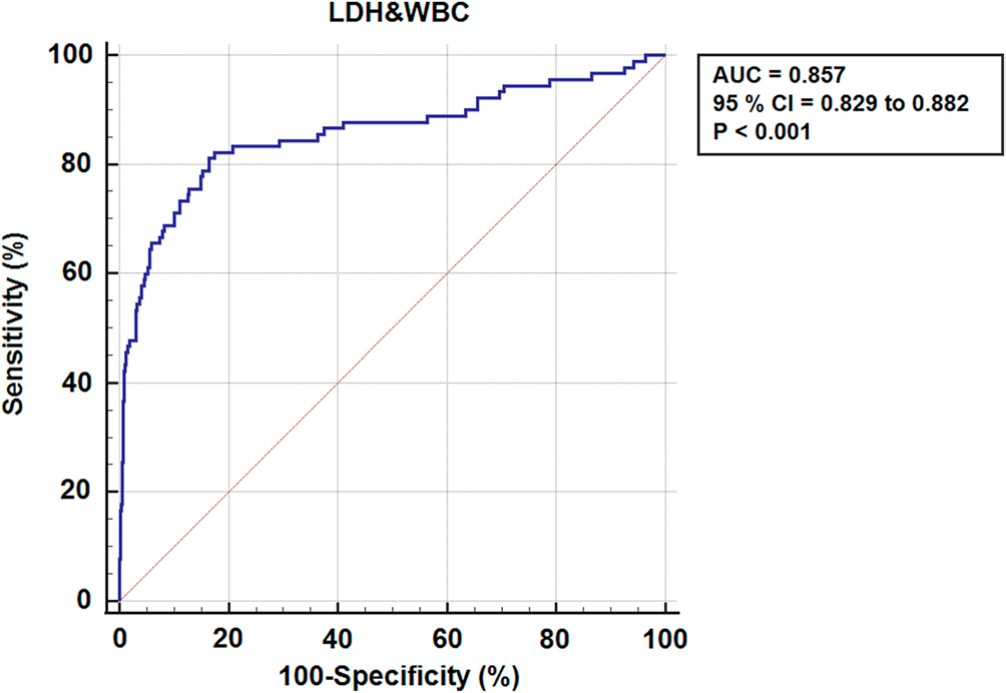
ROC curve for the combination of LDH and WBC.

In addition, the study revealed the ROC curves for the WBC and LDH predictors and provided detailed information in Fig. 2 and Fig. 3. The predictors LDH (p < 0.001, AUC = 0.800, 95%CI (0.769 to 0.829)) and WBC (p < 0.001, AUC = 0.774, 95%CI (0.796 to 0.829)) had cut-off values of 266.26 U/L and 11.7 × 10^9^ cells per liter, respectively. The combined factors significantly increased the AUC area, indicating a better diagnostic value. The highest diagnostic value of 0.850 was achieved with the combination of LDH and WBC, as shown by the area under the ROC curve in Fig. 4. Overall, these findings provide valuable insights into the risk factors and predictors of ACS in patients with tibial diaphysis fractures.

## Discussion

Tibial diaphysis fractures are common long bone injuries that occur at varying rates in males and females of different age groups^1^. The AO classification type 42-A3 is the most prevalent fracture type, with causes including falls, sports-related activities, and road traffic accidents^4–6^. Patients with these fractures may experience tenderness and, in some cases, a visible deformity. Tibial diaphyseal fractures are the leading cause of ACS, which poses a significant threat to limb integrity and requires immediate surgical intervention^9^. ACS in the lower leg occurs when increased pressure within a muscle compartment leads to reduced tissue perfusion, compromising motor and sensory functions^8,11^. Delayed intervention can result in prolonged hospital stays, increased expenses, and potentially severe outcomes such as amputation or death^12^. Risk factors for developing ACS in patients with tibial fractures have been discussed in the previous studies. However, the role of admission laboratory indicators in predicting ACS among these patients has been less explored, resulting in a gap in this research area. In line with evolving research, future studies should consider the prognostic value of inflammatory biomarkers from initial laboratory tests in ACS patients. Inspired by innovative approaches in orthopedic research, such as those suggested by Moldovan et al.^21^, identifying new biomarkers could significantly refine ACS risk assessment and management. This promising research area may lead to predictive models that enhance patient care through personalized treatment strategies.

This study represents a significant effort to explore potential risk factors that may influence the development of ACS in patients with tibial diaphysis fractures. Our findings identified several potential predictors of ACS through univariate analysis, including gender, referral patients, open fractures, crush injuries, hypoproteinemia, WBC, NEU, MON, EOS, LDH, and CK. Logistic regression analysis revealed that male patients, elevated WBC counts, increased LDH levels, and a history of crush injury were significantly associated with ACS development. In contrast, a history of falls on the same level and higher CHE levels were found to be protective factors against ACS in this patient group. Further analysis, such as ROC curve assessment, determined that the cut-off values for LDH and WBC in predicting ACS were 266.26 U/L and 11.7 × 10^9^ cells per liter, respectively. Notably, the most accurate diagnosis was achieved when both WBC and LDH were considered together.

Our study has identified gender difference as an independent risk factor for ACS, indicating that males have a significantly higher incidence rate of ACS compared to females, with a rate that is 3.200 times higher. Additionally, Obey et al. found that the risk of ACS was nearly 50% lower in female patients compared to male patients ^22^ and Aya et al.'s large-scale data analysis also yielded similar results, consistently showing that males face a higher risk of developing ACS ^23^. While some studies^24,25^ suggest that female patients have a similar risk of developing ACS after tibial fractures as male patients, our research, consistent with other studies^14,26^, has consistently shown that males face a higher risk. This may be attributed to the fact that males have a relatively larger muscle volume, which leads to thicker fascia and intermuscular septa due to a higher collagen content. Therefore, healthcare professionals should be aware of this gender difference and take it into account when assessing and managing patients with suspected or confirmed compartment syndrome. However, it is important to note that our study did not confirm previous findings^27,28^ that age was significantly younger in the ACS group compared to the non-ACS group. One possible reason for this discrepancy could be that our study excluded patients under 18 years old, which could have influenced the results.

Crush injuries, known for their potential to inflict considerable tissue damage and destruction, are commonly categorized as high-energy injuries; in contrast, falls occurring on the same level are undeniably viewed as a manifestation of low-energy injuries^29,30^. Crush injuries frequently transpire in areas of the body that are susceptible to being trapped or compressed by heavy objects, machinery, or vehicles. Such areas include the limbs, hands, chest, and abdomen. Our study has shown that patients who suffered from tibial diaphysis fractures caused by crushing injuries were 4.622 times more susceptible to developing ACS compared to those who experienced ACS from other types of injuries. Interestingly, our findings suggest that falls on the same level may offer a certain degree of protection against ACS. In their meta-analysis of nearly 50,000 patients, Mortensen et al. found a clear and direct link between high-energy trauma and the incidence of ACS^31^. Additionally, other study conducted by Wang et al. have also demonstrated a strong association between high-energy trauma and the development of ACS^32^. Our study's results are in agreement with the findings of these researchers, suggesting that high-energy trauma can lead to the development of ACS. The mechanism behind this correlation is believed to be related to the amount of energy that is initially dissipated in the soft tissues during the trauma, which can subsequently cause ischemia and tissue damage, leading to the onset of ACS^28^. In addition, it should be noted that the expansion of damaged tissue resulting from acute crush injuries or ischemic reperfusion can lead to intracompartmental swelling, which is a key factor in the development of ACS. It is worth noting that some studies have reported conflicting findings regarding the association between high-energy trauma and the development of ACS^13,33^ with some studies even suggesting that low-energy trauma may also be associated with its development^34^. One potential explanation for the conflicting findings regarding the association between high-energy trauma and the occurrence of ACS is the challenge of accurately assessing the amount of energy released during the initial trauma. This determination can be difficult due to limitations in medical record reviews, which may not provide a complete understanding of the injury mechanism, including important details such as the cause and location of a crush injury.

LDH, which is present in both the plant and animal domains, plays a critical role in the anaerobic metabolic pathway and is frequently utilized in the diagnosis of muscle-related disorders such as muscle necrosis and rhabdomyolysis^35^. In our study, we have discovered a potential correlation between the levels of LDH in serum and the occurrence of ACS. Specifically, our findings suggested that patients with higher levels of LDH were at a slightly greater risk of developing ACS compared to those with lower LDH levels (OR = 1.003). Our study was the first to investigate the potential correlation between elevated levels of LDH and the occurrence of ACS, as no prior research had been conducted on this relationship. Interestingly, we drew some inspiration from the study conducted by Vrouenraets et al., who suggested that severe limb toxicity can lead to compartmental compression syndrome and severe rhabdomyolysis. They also found a linear correlation between the level of LDH and toxicity grade, which is consistent with our study results and supports the potential relationship between elevated levels of LDH and the occurrence of ACS^36^. Additionally, we have identified a cut-off value of 266.26 U/L for LDH using ROC curve analysis. When muscles are injured, cell membranes can rupture and release intracellular enzymes such as LDH, causing their levels to rise in the bloodstream. Additionally, tissue necrosis and inflammatory responses can cause local tissue swelling and increased pressure, ultimately leading to compartment syndrome. While our study provides some clues, further research is needed to fully confirm the mechanisms underlying this relationship.

The rise in pressure within the compartment is widely recognized to create a hypoxic and ischemic microenvironment, ultimately exacerbating muscle necrosis and causing aseptic inflammation. These changes inevitably lead to alterations in the immune cell profile, as immune cells respond to and are affected by the inflammatory environment^37–39^. Our study aimed to investigate the role of immune cells in the development of ACS and found a significant increase in the level of WBC in the ACS group compared to the Non-ACS group. Through ROC curve analysis, we have identified that patients with a WBC count above 11.7 × 10^9^ cells per liter have a relatively higher risk of developing compartment syndrome. Similar to the characteristics of LDH we previously described, an elevated WBC count could also be a predictor of impending limb toxicity. This limb toxicity can, in turn, lead to ACS^36^. As we are aware, WBC serve not only as potent antimicrobial agents but also play a crucial role in the removal of necrotic tissue^40^. Consequently, the WBC levels can, to a certain extent, indicate the severity of tissue necrosis, which is worth noting as a key characteristic of ACS. Furthermore, Tollens et al.^41^ have found that NEU, a type of WBC, may exacerbate tissue injury and edema in the reperfused muscle after migrating to the affected area. By monitoring WBC count, doctors can take preventive measures and increase monitoring frequency in at-risk patients. In conclusion, the potential use of WBC count as a predictor for Compartment Syndrome is an intriguing prospect that warrants further investigation.

CHE is crucial in the termination of synaptic transmission as it hydrolyzes the neurotransmitter acetylcholine, thereby ensuring proper communication between neurons^42^. In our research, we have discovered that an increase in cholinesterase levels plays a suppressive role in the development of compartment syndrome. This suggests that low levels of cholinesterase are associated with the occurrence and progression of compartment syndrome. The case reports by Bala et al. have drawn similar conclusions to ours: patients with organophosphate poisoning who inevitably experience a decrease in CHE levels develop ACS^43^. The possible reasons for this phenomenon are that lower CHE levels are associated with reduced neuromuscular junction function and decreased muscle activity, eventually leading to an increased risk of ACS^44^. The correlation between CHE and compartment syndrome provides us with the insight that monitoring CHE levels can potentially aid in predicting the occurrence of compartment syndrome. Therefore, it may be beneficial to include CHE level monitoring in the diagnostic and management protocol of patients at risk of developing compartment syndrome. However, further studies are needed to confirm the relationship between CHE levels and compartment syndrome and to establish the clinical significance of monitoring CHE levels in patients at risk of developing compartment syndrome.

While this study represents an important initial investigation into the risk factors for ACS in patients with tibial diaphysis fractures, focusing on admission laboratory examinations, there are several key limitations that must be acknowledged. First, as this was a retrospective study, we could not collect all potential variables related to ACS development, such as the specific fracture types, which might differentially impact ACS risk. Our study may also have overlooked important confounding factors, introducing residual confounding that could influence the observed associations. Second, we depended on self-reported patient-specific variables, including smoking, drinking, and medical comorbidities, which may lead to inaccuracies or inconsistencies in the data. This is a typical limitation in retrospective studies that rely on medical records or patient surveys. Third, our study's relatively small sample size increases the likelihood of random errors and limits the power of statistical analyses. A larger sample size and a prospective design would enhance the robustness and reliability of our results. Fifth, the retrospective nature of this study and inherent variations in clinical practice led to a lack of standardization in the diagnosis of ACS across cases, which is a limitation we openly acknowledge. Such diagnostic variability could influence the interpretation of our results and the accuracy of our data analysis. While all included patients were diagnosed with ACS, not every individual underwent compartment pressure measurements, which would have provided a more objective and standardized diagnostic approach. This inconsistency in diagnostic methods may affect the reliability of our findings. Besides, we did not find specific objective pressure values in our retrospective analysis of patient data, even though compartment pressure measurements were performed for some patients (we learned from consulting the attending physicians at the time that they all met the standard criteria). Furthermore, we acknowledge the omission of a comprehensive assessment of the patients' injury severity, such as through the Injury Severity Score (ISS), in our study. The absence of such an evaluation might have influenced the systemic measured parameters, as injury severity alone can contribute to their elevation. We regret not incorporating this critical aspect into our data analysis and recognize it as an area for improvement in future research endeavors.

In summary, our study has shown that male patients, elevated WBC counts, increased LDH levels, and a history of crush injury were independent predictors of ACS in patients with tibial diaphysis fractures. The cut-off values for LDH and WBC to predict ACS are 266.26 U/L and 11.7 × 10^9^ cells per liter, respectively. The findings underscore that while clinical evaluation remains fundamental in the initial assessment and suspicion of ACS, laboratory tests, particularly the levels of lactic dehydrogenase (LDH) and white blood cell (WBC) count, provide quantifiable markers that significantly enhance the predictive accuracy for ACS. The established cut-off values for LDH and WBC, derived from our analysis, offer clinicians practical tools for a more nuanced risk stratification, facilitating timely and targeted interventions. Therefore, the impact of our study is most pronounced in integrating clinical acumen with laboratory evidence to refine ACS risk assessment, ultimately aiming to improve patient outcomes through informed clinical decision-making. In addition, we have identified a history of falls on the same level and higher CHE levels as protective factors against ACS in this patient population. It is important to acknowledge that our study had some limitations, particularly its small sample size, which may have affected the generalizability of our results. Therefore, it is crucial to conduct larger studies to confirm our findings and further investigate other potential risk factors for ACS in this patient population. Overall, our findings, emphasizing the importance of admission laboratory examinations, offer a foundation for clinical doctors to evaluate the risk of ACS in patients with tibial diaphysis fractures and implement targeted therapies accordingly. However, we recognize that more research is needed to fully understand the complex interplay between various factors and their impact on the development of ACS.

## Data Availability

The data that support the findings of this study are available from Baoding First Central Hospital, but restrictions apply to the availability of these data, which were used under license for the current study, and so are not publicly available. Data are however available from the authors upon reasonable request and with permission of the corresponding author of our article (Yubin Long, E mail : longyubin1987@163.com).

## Abbreviations

ACS: Acute compartment syndrome
ROC: Receiver operating characteristic
AUC: Area under the curve
BMI: Body mass index
WBC: White blood cell
NEU: Neutrophil
LYM: Lymphocyte
MON: Monocyte
EOS: Eosinophil
BAS: Basophil
RBC: Red blood cell
HGB: Hemoglobin
HCT: Hematocrit
MCV: Mean corpuscular volume
MCH: Mean corpuscular hemoglobin
MCHC: Mean corpuscular hemoglobin concentration
PLT: Platelet
MPV: Mean platelet volume
DBIL: Direct bilirubin
GGT: Gamma glutamyl transpeptidase
TP: Total protein
ALB: Albumin
GLOB: Globulin
UREA: Ureophil
UA: Uric acid
LDH: Lactic dehydrogenase
CK: Creatine kinase
TG: Triglyceride
TCO_2_: Total carbon dioxide
CHE: Cholinesterase
ALP: Alkaline phosphatase
OSM: Osmotic pressure
